# When and where mortality occurs throughout the annual cycle changes with age in a migratory bird: individual vs population implications

**DOI:** 10.1038/s41598-019-54026-z

**Published:** 2019-11-22

**Authors:** Fabrizio Sergio, Giacomo Tavecchia, Alessandro Tanferna, Julio Blas, Guillermo Blanco, Fernando Hiraldo

**Affiliations:** 1 Department of Conservation Biology, Estación Biológica de Doñana - CSIC, C/Americo Vespucio 26, 41092 Seville, Spain; 2 Animal Demography and Ecology Unit (GEDA), IMEDEA (CSIC/UIB), C/M. Marques 21, 07190 Esporles, Spain; 3 Department of Evolutionary Ecology, Museum of Natural Sciences, CSIC, C/José Gutiérrez Abascal 2, 28006 Madrid, Spain

**Keywords:** Animal migration, Population dynamics

## Abstract

The annual cycle of most animals is structured into discrete stages, such as breeding, migration and dispersal. While there is growing appreciation of the importance of different stages of an organism’s annual cycle for its fitness and population dynamics, almost nothing is known about if and how such seasonal effects can change through a species lifespan. Here, we take advantage of the opportunity offered by a long-term satellite/GPS-tracking study and a reliable method of remote death-detection to show that certain stages of both the annual and life cycle of a migratory long-lived raptor, the Black kite *Milvus migrans*, may represent sensitive bottlenecks for survival. In particular, migratory journeys caused bursts of concentrated-mortality throughout life, but the relative importance of stage-specific survival changed with age. On the other hand, the balance between short-stages of high mortality and long-stages of low mortality made population-growth similarly dependent on all portions of the annual cycle. Our results illustrate how the population dynamics of migratory organisms can be inextricably linked to ecological pressures balanced over multiple stages of the annual cycle and thus multiple areas of the globe, suggesting the frequent need for challenging conservation strategies targeting all portions of a species year-round range.

## Introduction

There is growing appreciation of the importance of different stages of an organism’s annual cycle for its fitness and population growth^1,2^. Such appreciation may especially affect migratory species that use far away sites during different portions of the year and that are increasingly reported to decline for unknown causes^3–5^. In particular, separate stages of the annual cycle may impose different threats and pressures on organisms: for example, migration can expose animals to hostile weather and a wide range of anthropogenic risks along the route, while the breeding season may affect body condition as a consequence of intensive reproductive effort. As a result, an increasing number of so-called “full annual cycle studies” has reported how population abundance and key vital rates can be limited by factors operating in specific periods of the year e.g.^6–9^.

On the other hand, while the importance of certain stages of the year is increasingly recognized, an aspect of full annual cycle research that is virtually unknown is whether and how these seasonal effects change as individuals progress through their lives. Incorporating a full life cycle perspective into full annual cycle studies seems critical, because age-structure in vital rates is a fundamental determinant of individual fitness and population growth^10,11^. This is especially so for long-lived species, which are subject to threats that may change through life, as individuals proceed from initial juvenile stages, to immature non-breeding phases, to adult breeding careers and to the final, senescent portions of their life. For these species, identifying sensitive bottlenecks in both their annual cycle and life cycle is fundamental for efficient management and conservation^12^.

Overall, progress in this area of research has been slow, mainly because of the challenge of obtaining comprehensive data covering all stages of the annual cycle e.g.^13^, let alone to couple it with comprehensive coverage of a species lifespan. As a consequence, the development of full annual cycle biology has been identified as a major research priority^2^. To date, full annual cycle studies have been based on four main methodologies: (1) direct sampling of some stages of the annual cycle to indirectly estimate the figures for other ones (e.g. non-stationary, migratory stages), based on a number of assumptions e.g.^9,14,15^; (2) relating annual parameters (e.g. annual survival) to environmental conditions in different periods of the year to indirectly infer which stage of the annual cycle could be more limiting e.g.^16,17^; (3) implementing full annual cycle models, where missing estimates for some stages were borrowed from other studies, gathered through expert opinion, or modelled as a range of likely values^18,19^. (4) Finally, recent technological advances in biologging are allowing the unprecedented option of remotely monitoring the movements and survival of individuals throughout the year. However, the high costs involved have implied limited sampling, and the outcome estimates lie on two assumptions: that tagging has no impact, and that real deaths can be separated from cases of transmitter-failure (reviewed in^20^). To our knowledge, no full annual cycle biologging-study has provided such tests, and deaths have often included dubious cases classed as “probable deaths”, with authors admitting a degree of subjectivity in their assignments e.g.^21^. Given the above, current knowledge incorporates a margin of uncertainty caused by indirect or borrowed estimates, unsure death assignments, pooling of different stages into coarse-level categories (e.g. a month that includes two different stages), or sampling of a limited portion of a population (only juveniles, only territorial adults, or only individuals above a certain weight to avoid excessive transmitter-loads)^14–21^. Thus, while much progress has been made, there is a need for additional robust studies based on detailed estimates for all stages of the annual and life cycle, in order to complete our picture of seasonal effects.

Here, we exploit the opportunity offered by a long-term satellite GPS-tracking study and a new method of remote death-detection to examine for the first time how variation in vital rates through all stages of the annual cycle and life cycle may affect the population dynamics of a migratory, long-lived raptor, the Black kite *Milvus migrans*. Below, for simplicity, we define 1-year olds as “juveniles” and 2–6 years olds as “young adults”. Also, because the study included non-breeding individuals, to avoid confusion we mainly refer to the post-breeding migration as “southward migration” and to the pre-breeding migration as “northward migration”.

## Results

**Figure 1:**
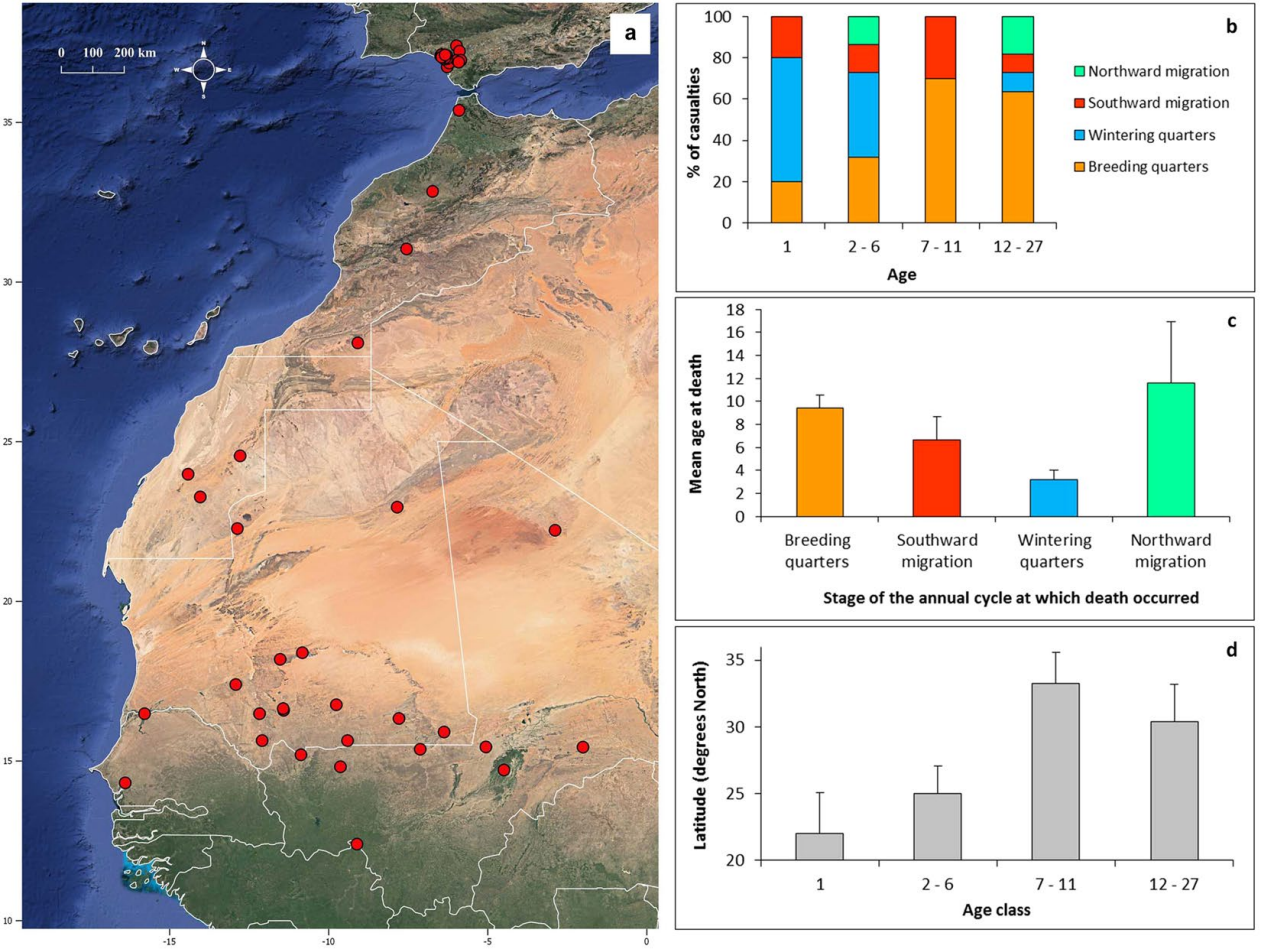
Characteristics of casualties of satellite/GPS-tagged kites. Death sites covered the whole annual range of the species, including the Spanish breeding quarters, the African wintering quarters and the regions covered by the migratory journeys (panel 1a). The percentage of casualties that occurred in each stage of the annual cycle varied through life (1b), so that mean age at death varied with the stage of the annual cycle in which an individual died (1c), while individuals of different ages died on average at different latitudes (1d). Error bars represent 1 se. The map was created through QGis Desktop 3.6.0 (http://qgis.osgeo.org).

Satellite-GPS tagging uncovered 53 casualties (Fig. 1a): 43% occurred in the Spanish breeding quarters, 30% in the African wintering quarters, 17% in the post-breeding migration and 9% in the pre- breeding migration. These figures changed with age (χ^2^_3_ = 11.9, P = 0.008; Fig. 1b): death occurred more frequently in the African wintering quarters for juveniles and young adults (χ^2^_2_ = 10.7, P = 0.001), while it switched to become more frequent in the breeding quarters after 7 years old (χ^2^_2_ = 7.7, P = 0.006). As a result, age at death varied significantly among kites that died in different stages of the annual cycle (ANOVA, F_3,49_ = 4.47, P = 0.007; Fig. 1c): on average, individuals that died while wintering in the African quarters were younger than those dying in other stages, particularly in the pre-breeding migration. Similarly, the latitude at which kites died varied through life (ANOVA, F_3,49_ = 3.34, P = 0.027; Fig. 1d): on average, sites of death were below 25° of latitude (below Northern Mauritania) for kites that died early in life as still juveniles or young adults, but above 30° (above southern Morocco) for kites that died after 7 years old. While 64% of the casualties that occurred on migration were concentrated in the Sahara Desert, the spatial distribution of death sites was never clustered at specific locations (z score ≥ 0.52 within each stage of the annual cycle).

**Figure 2:**
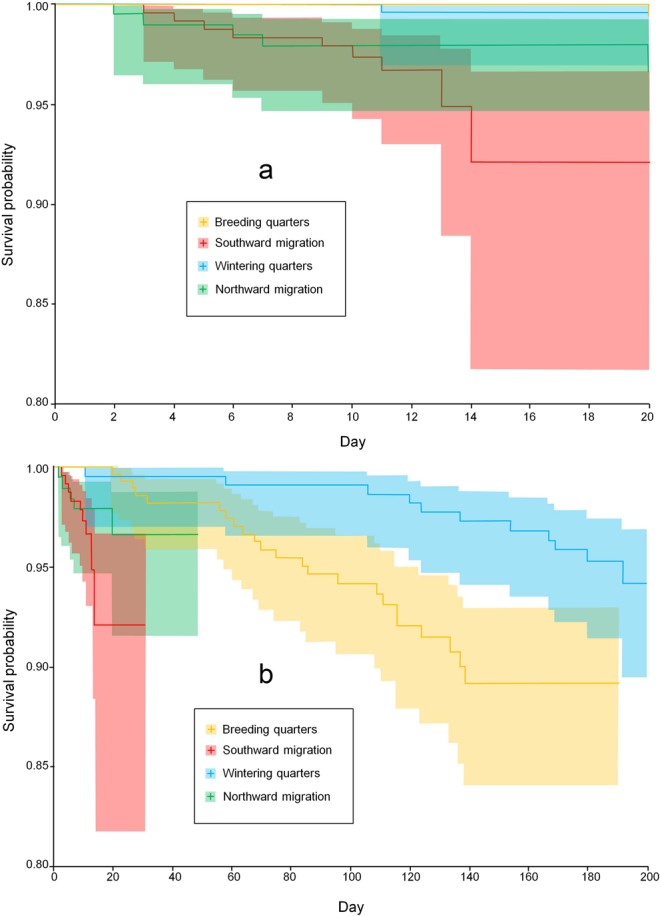
Survival probabilities of satellite/GPS-tagged kites for different stages of the annual cycle. The relative risk of death was highest during the southward migration, followed by the northern migration, the breeding season and the African wintering stage. To avoid visual clutter, we show separately the first 20 days of exposure (panel a) and the overall period of exposure (panel b). Coloured bands around each line represent 95% confidence intervals. A version of the same figure but without coloured bands is available in Supplementary Fig. S.1.

**Figure 3:**
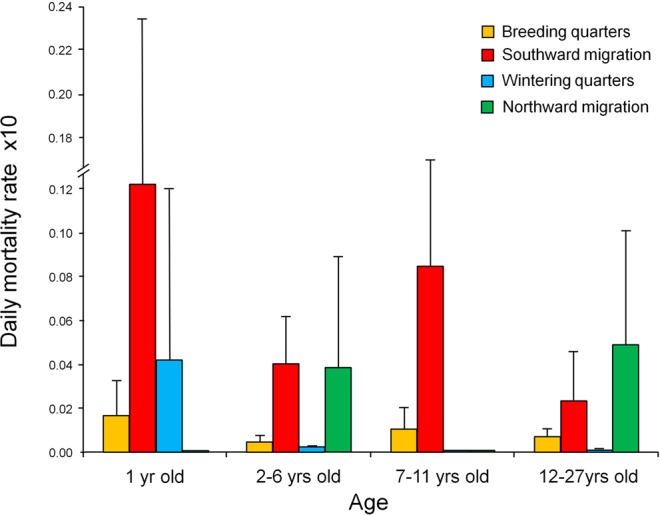
Daily mortality rates of satellite/GPS-tagged Black kites for different stages of the annual cycle. Estimates were assessed through Kaplan-Meier procedures and multiplied by 10 for clarity of presentation, in order to avoid excessive numbers of zero-decimals. Error bars represent 1 se. The sample of tracked individuals for each bar is (from left to right): 32, 18, 16, 2, 54, 49, 52, 45, 30, 22, 19, 17, 25, 24, 24, 24.

When controlling for the differential duration of each stage of the annual cycle through Kaplan-Meier models, daily mortality rates were highest during migrations, particularly the post-breeding journey, intermediate in the Spanish breeding quarters, and lowest in the African wintering quarters (Fig. 2). Once again, this pattern varied as individuals progressed through life: the interaction between age and stage of the annual cycle was significant (Likelihood ratio test = 11.8, P = 0.008). In particular, daily mortalities, as estimated by Kaplan-Meier estimators, were highest in the post-breeding migration and in the wintering African quarters for juveniles, in both migration-journeys for young adults, in post-breeding migration for individuals in prime age, and in both migrations for senescent kites (Fig. 3).

**Figure 4:**
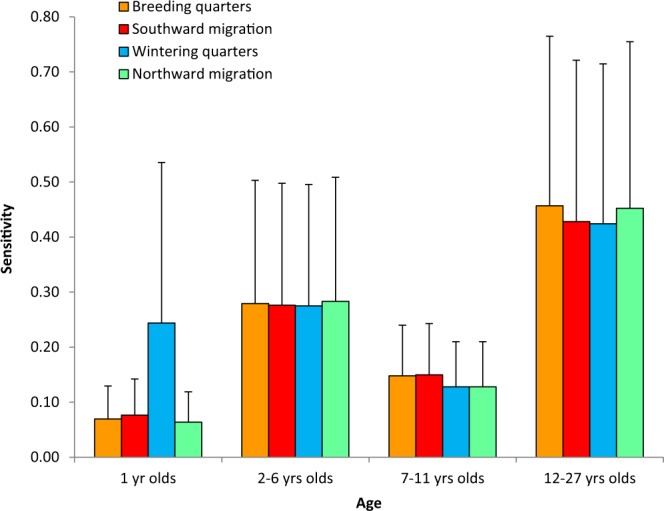
Sensitivity of population growth rates to variation in mortality rates across stages of the annual cycle and across stages of the life cycle of satellite/GPS-tagged Black kites. The error bars represent uuncertainties in sensitivity values (1 se), calculated by bootstrap from 1000 population matrices with parameter values randomly taken from their respective estimated distributions.

Sensitivity analysis, which weighed both mortality rates and the period of exposure over which they operated, showed that population growth rate was more sensitive to variation in survival across age classes than across stages of the annual cycle (Fig. 4, Supplementary Table S.2). Population growth mainly depended on survival of young adults and older kites, as already known for this population (Sergio *et al*. 2011a). Over these ages, all stages of the annual cycle were similarly important for population growth. The latter was poorly dependent on breeding performance, as typical of long-lived species (Supplementary Table S.2).

## Discussion

While the significance of seasonal effects on animal ecology is increasingly appreciated^2^, here we expand previous studies by showing how the relative importance of different stages of the annual cycle changes as individuals progress through life. In particular, stage-variation interacted with age in two main ways. Firstly (1), seasonal effects operated differently for juveniles than for older individuals (Fig. 3). In our population, nearly 60% of kites die during their first year of life^22^ and here we show that this severe bottleneck, which rapidly shrinks the available pool of adults, is mainly concentrated in the first southward migration and in the protracted stay in the African wintering quarters. Thus, factors operating far away from the breeding quarters will initially shape the type of individuals that will compose the eventual breeding pool. Secondly (2), the relatively rapid migrations concentrated casualties at all ages, but the relative importance of the pre-breeding vs post-breeding migration varied through life (Fig. 3, see below). Migrations are known to entail higher costs and risks than more static stages, as a consequence of metabolic exhaustion, navigation errors and unpredictable exposure to predation, anthropogenic threats and adverse weather (review in^23^). As a consequence, the few studies that managed to directly or indirectly estimate survival on migration have typically reported higher mortality en route than in the other stages of the annual cycle^7,9,14,15,21,24^. Our results broaden this picture by showing that migration-mortality varied through life: it was especially acute in juveniles, consistently more elevated than in other stages for all ages, but more pronounced in the southward migration than in the pre-breeding journey for certain age-classes. These results have major implications for the ecology of migratory species: first, besides the above mentioned severe bottleneck for juveniles, migration continued to be a recurrent mortality-filter through the whole lifespan of an individual. Thus, migration was an underlying pressure that “permeated” the whole annual and life cycle, rather than acting solely as an initial filter that selected a set of superior individuals subsequently freed from migratory costs. Second, after the first year of age, mortality was extremely low in the African wintering quarters. Thus, migration could be seen as a short, risky investment to conquer a long-lasting, low-mortality “safe haven”. Under this scenario, whether a population evolves or maintains migratory behaviour will likely depend on the balance between the fitness costs and benefits operating in static and non-static periods of the annual cycle, once integrated throughout life. For most species studied so far, factors deteriorating the already high mortality rates en route could affect the long-term profitability and maintenance of migration itself. In turn, this makes migrants particularly susceptible to threats operating along their travel routes (e.g. habitat loss at stopovers, or climate change affecting wind support).

An interesting aspect of our results was the age-related differences in mortality between the southward and northward migration. In particular, the southward migration imparted a higher death toll on juveniles and 7–11 years olds than on other age-classes (Fig. 3). Inexperienced juveniles on their first migration have been shown to be less proficient than adults, often choosing unfavourable weather conditions for travelling, suffering more their adverse effects, and advancing more slowly with higher energy expenditures^25–28^, while their inefficiency and wrong decisions during migration have been directly implicated in their deaths^28,29^. The mortality peak for 7–11 years olds was less expected, because this is the prime age of maximum survival and reproduction^22^. Their higher mortality in the southward migration may represent a carry-over, delayed cost of reproduction for the following reasons: (1) individuals that died on migration at this age had raised more than double the number of fledglings than individuals that did not die on migration at this age (authors’ unpubl. data); (2) in this population, seasonal drought progressively worsens food availability through the breeding cycle and the body condition of breeders deteriorates in parallel, to reach a minimum peak just before the southward migration^30,31^. The latter could thus represent a filter that removes individuals incapable to match their breeding investment to available resources while maintaining adequate body condition. More study is currently under way to clarify this point.

A further intriguing result was the generally higher mortality rates imparted by the southward than the northward migration. Pre-breeding migrations are generally considered more challenging because of the urgency to arrive earlier than others at the reproductive quarters^23,32^. Also, in the western Afro-Palearctic migration system framing our study, weather conditions (e.g. wind and thermal support) are more adverse during the northward migration^23,33^. In agreement with the above, previous full annual cycle studies have usually reported higher mortalities in the pre-breeding than in the post-breeding migration^7–9^. We believe that the lower mortality that we observed in the northward journeys was not indicative of easier conditions, but rather caused by counter-strategies that minimized its higher danger. Compatibly with this idea: (1) juveniles almost systematically avoided the northward migration; (2) several young individuals started to migrate north, but quickly aborted the intent, as if encountering excessive difficulties, which was never observed in southward journeys; (3) young adults only migrated very late, once conditions were more favourable; even so, they suffered weather-effects more than adults, suggesting they could have suffered higher survival costs if they had migrated earlier; (4) adults were more independent of wind-support, but the time spent travelling and at stopovers was 2–7 times longer in the northward than southward migration; (5) for all ages, components of the northward migration, but none of the southward migration, affected long-term recruitment, breeding performance, annual survival and longevity^26,34^. All this suggests that mortality costs of the northward migration may have been paid over longer periods rather than instantly, as a form of carry-over effect^35^, while active strategies to avoid excessively dangerous journeys may have further canalized short-term survival over such sensitive stage of the annual cycle e.g.^36^. These considerations illustrate the interconnectedness of different stages of the annual cycle and the challenges involved in identifying the most sensitive ones.

While mortality per unit time uncovered the prospects and risks faced by an individual during each stage of the annual cycle, sensitivity analysis incorporated the duration of each stage to examine the population-level consequences of seasonal mortalities. Once stage-duration entered the picture, all stages became similarly important for population growth (Fig. 4). This is because low mortality in certain stages was counterbalanced by their long-duration, leading to the removal of similar portions of the population as shorter stages with more severe mortalities. This illustrates the importance of not automatically equating variation in seasonal survival with population limitation: survival prospects may affect individual-level risk perception, behavioural strategies and life history decisions, while population-impacts will incorporate the complex interaction between vital rates, stage duration and carry-over effects, a conceptual dichotomy that has received very limited appreciation. Whatever the mechanism behind it, the fact that population growth was similarly sensitive to all stages implies serious challenges for management, because conservation will need to target all areas used by a species in its annual cycle, leading to the frequent need for international cooperation^3^. Finally, the population-implications of the trade-off between seasonal mortality rates and stage duration imply that factors increasing mortality during the longer stages of the annual cycle (e.g. at the breeding or wintering quarters) will require special monitoring, as these could have a high potential for triggering fast declines e.g.^1^.

In conclusion, migratory species live complex, dynamic lives, often structured around the need to reach safe sites by overcoming lifelong, periodic mortality-bottlenecks that recurrently and rapidly filter out part of the population. While certain stages of the annual cycle may shape which phenotypes progress through life, all stages are likely to contribute to long-term population maintenance. Thus, investigating or managing one stage of the annual or life cycle in isolation is unlikely to attain proper insight or conservation. Predicting the consequences of global or climate change will be challenging for migratory species, especially when these affect different regions in different ways, but factors that will raise mortality rates indiscriminately over all stages (e.g. any unfavourable form of large-scale global change), or during the longest stages of the annual cycle will be priority candidates as drivers of population declines.

## Methods

### Model species

The Black kite is a medium-sized, migratory, long-lived raptor. Its annual cycle is composed of a breeding season (usually spanning March-August), and a non-breeding period spent in the African wintering quarters (September-February) connected by pre- and post-breeding migratory journeys of 8–35 days^26,34^. The life cycle can be organized into the following well-recognized sequential stages, based on the attainment of breeding status and the start of senescence, corresponding to variations in breeding performance and survival^22,30,34,37,38^: (a) age 1 (juvenile individuals that never breed and mostly remain in Africa all year round; this is the major age-bottleneck, with mortality removing nearly 60% of the population); (b) age 2–6 (young adults attempting to establish a territory or during their initial, usually unsuccessful breeding attempts); (c) age 7–11 (prime age of maximum breeding and survival rates); and (d) age 12–28 (senescent stage with lower breeding and survival rates).

### Field procedures

Stage-specific data on mortality were available through satellite/GPS-tagging for 108 kites of known age, selectively trapped between 2007–2014 through more than 1,400 trapping hours so as to cover the whole lifespan of the species (from fledgling to 27 years old, details in^26^). Kites were tracked by GPS-satellite tags (model PTT-100 Solar Argos/GPS PTT, manufactured by Microwave Telemetry, Inc.). The transmitters weighed 22 grams (<3% of the body mass) and were powered by solar panels, allowing a lifespan of 3 years declared by the manufacturer, but some were still functioning after five years^20^.

### Statistical analyses

Confidence in the usage of GPS-tags to investigate mortality-rates can be improved by the satisfaction of two conditions. First, survival should not be impaired by tagging: in a previous assessment, comparison of the vital rates of marked kites with those of control individuals of comparable age and status showed no effect of satellite-tagging on survival, recruitment, longevity, breeding performance, as well as offspring provisioning, condition and physiological stress^39^. Second, casualties should be reliably separated from cases of transmitter-failure. We have recently shown^20^ that engineering and location data from our tags can reliably identify deaths and separate them from cases of radio-failure on the basis of three simple indicators: (1) stationarity of GPS locations; (2) confirmation or disproof of stationarity by background Doppler data from the Argos System; and (3) indication of transmitter malfunctioning by low frequencies of GPS-locations preceding signal loss (see details in^20^). This classification key was capable of correctly classifying 100% of all cases of sure deaths (individuals physically recovered as dead) and 100% of sure transmitter-failures (individuals subsequently observed alive) in both a model-building and a validation dataset based on data from three different species^20^. Satisfaction of both conditions implied that, here, we could remotely examine variation in mortality rates through multiple continents and across all stages of the annual cycle and of the lifespan of a long-lived species, with lower levels of statistical noise generated by uncertain deaths and transmitter-impacts. However, we still express some caution as we cannot discount the possibility that tagging somehow altered the seasonal distribution of mortalities.

Several methods can be used to estimate survival from known-fate data. We used Kaplan-Meier estimators with right-censored data e.g.^40,41^ to estimate the daily survival probabilities of kites in relation to stages of the annual cycle, *k*, and of the life cycle, *j*. In doing so, we assumed that an animal exiting a period alive would enter the following period as a new individual. For clarity of explanation, below we refer to the stages of the annual cycle as “stages” and to the stages of the life cycle as “age-classes” or “ages”. Each death event(s), *n*, set the interval *t*, over which we calculated the survival probability S_j,k,*t*_, as S_j,k,*t*_ = (r_j,k,*t*_ − *n*)/r_j,k,*t*_, where r_j,k,*t*_ is the number of animals of age-class *j* in stage *k* at risk of death during *t* (Fig. 2). We then scaled all S_*t*_ values to obtain a daily survival, dS_*t*_ which is comparable across all age-by-stage combinations. For each of these combinations, to obtain an estimate of the average daily survival, we took the geometric mean of these values and combined their variances using the δ-method (see^42^). Note that, because estimate-precision depends on the number of animals at risk of death at the beginning of the period, we eliminated those *t-*intervals with 5 or less kites at risk. Finally, for each age-class we calculated the stage survival as ϕ_*j.k*_ = $\widehat{dS}\,$_*j,k*,_^*l*^, where *l* is the duration of the stage for that age-class. In three out of the sixteen stage-by-age combinations (4 stages by 4 ages), mortality events did not occur and we assumed a survival probability of 1. Because significance-testing through Kaplan-Meier estimators requires larger samples than those available here or in almost any tracking study^43,44^, we tested the significance of age, stage and their interaction through the following procedure: first of all, the dataset comprised 53 individuals that died and 52 individuals that never died during the course of their satellite-monitoring (three individuals were not employed in this analysis because tracked for too short). These 52 individuals were used as control individuals and we randomly selected a random day of their tracking period as their simulated day of death. The age and stage of the annual cycle of such 52 simulated death events was then compared to the age and stage of the 53 actual deaths by means of a GLM with binomial errors and logit link function^45^.

In order to investigate how variation in vital rates along the life cycle contributed to population growth, we combined ϕ_*j.k*_ values into an age-structured population matrix model^11^ in which the annual survival of each age-class, *j*, was expressed as the product of the respective ϕ_*k*_ survival probabilities. Age-dependent natality values for the matrix model were taken from^22^ and the duration of each stage is reported in Supplementary Table S.1. Sensitivity values and their uncertainties were calculated by bootstrap from 1000 population matrices with parameter values randomly taken from their respective estimated distributions^12^.

We used the “Average Nearest Neighbor” tool of ArcGIS 10.6 to test whether locations of death sites were significantly clustered at specific areas. Departure from a random distribution was tested by means of a z-test^46^. Changes in the frequency of deaths events by age and stage of the annual cycle were tested by means of a χ^2^ test^47^. To avoid excessive numbers of cells with expected frequencies <5, for this analysis we pooled together all individuals of 1–6 years old and of 7–27 years old. Throughout, all models were implemented in R.3.5.0, all tests are two-tailed, statistical significance was set at α < 0.05, and all means are given ± 1 SE. Generalised linear models were built through R 3.4.4^48^ and Kaplan-Meier procedures were implemented through the package “survival”^49^. For simplicity, we define 1-year olds as “juveniles” and 2–6 years olds as “young adults”. Also, because the study included non-breeding individuals, to avoid confusion we mainly refer to the post-breeding migration as “southward migration” and to the pre-breeding migration as “northward migration”.

## Supplementary information

Supplementary information

## Data Availability

Data will be available on reasonable request from the authors.
