# Supplementary material for: When and where mortality occurs throughout the annual cycle changes with age in a migratory bird: individual vs population implications
Source: Sci Rep. 2019 Nov 22;9:17352. doi: 10.1038/s41598-019-54026-z (PMC6874661; doi:10.1038/s41598-019-54026-z)
Supplement: Supplementary file 1 — Supplementary information [file 41598_2019_54026_MOESM1_ESM.pdf]

When and where mortality occurs throughout the annual cycle changes with age in a migratory bird: individual vs population implications

Fabrizio Sergio, Giacomo Tavecchia, Alessandro Tanferna, Julio Blas, Guillermo Blanco and Fernando Hiraldo

Supplementary Figure S.1. Survival probabilities of satellite/GPS-tagged kites for different stages of the annual cycle. The relative risk of death was highest during the southward migration, followed by the northern migration, the breeding season and the African wintering stage. To avoid visual clutter, we show separately the first 20 days of exposure (panel a) and the overall period of exposure (panel b).

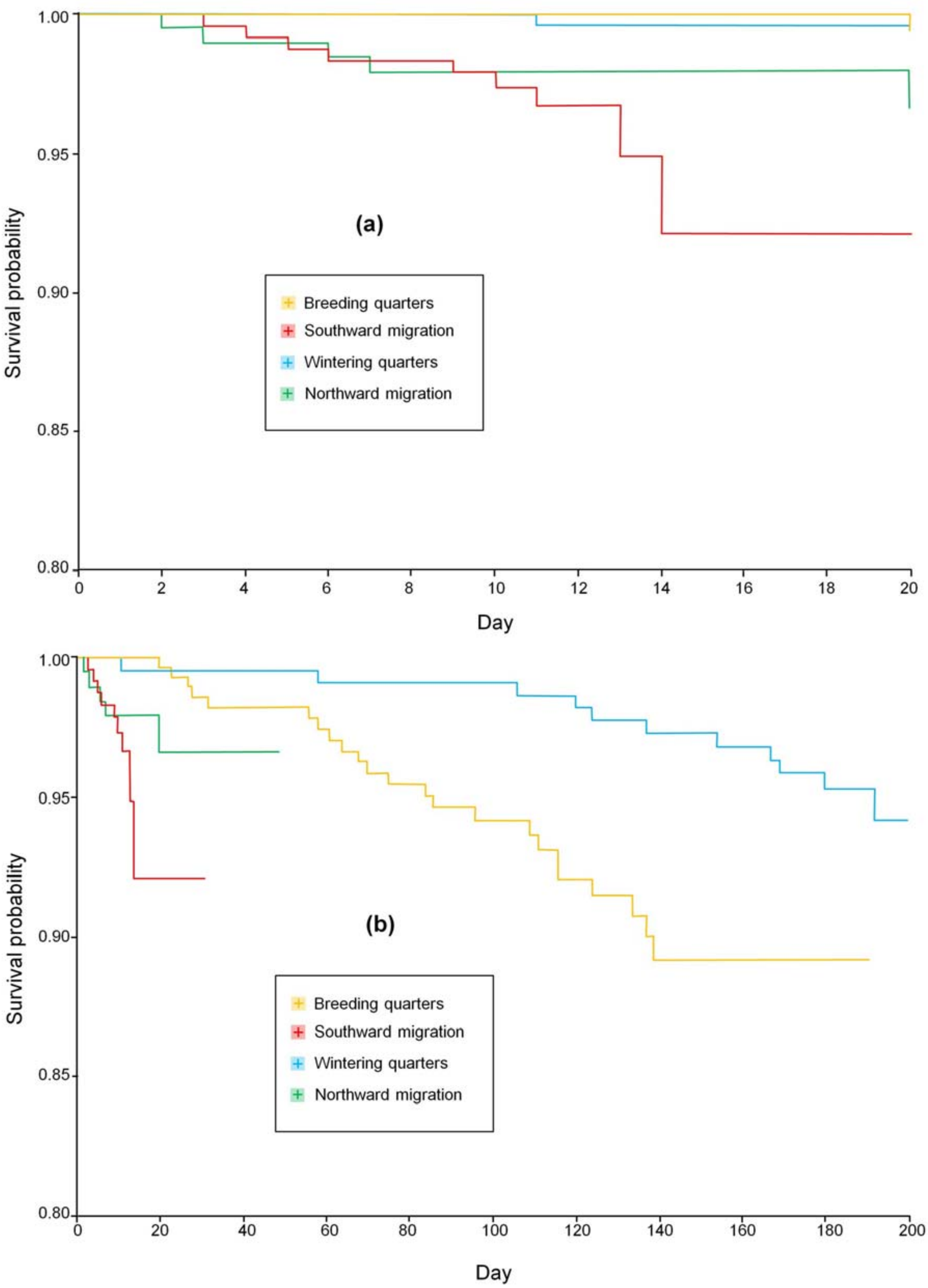

(a)
Breeding quarters
Southward migration
Wintering quarters
Northward migration
Survival probability
1.00
0.95
0.90
0.85
0.80
0
2
4
6
8
10
12
14
16
18
20
Day
(b)
Breeding quarters
Southward migration
Wintering quarters
Northward migration
Survival probability
1.00
0.95
0.90
0.85
0.80
0
20
40
60
80
100
120
140
160
180
200
Day

Supplementary Table S.1. Duration of each stage of the annual cycle of satellite/GPS-tagged Black kites employed in the age-structured population matrix model (see Methods). Durations are expressed as percentages of a full year.

Ageclass (years)	Stage of the annual cycle			
	Breeding grounds	Southward migration	Wintering stage	Northward migration
1	12.2	3.5	78.7	5.5
2-6	37.4	3.2	54.5	4.9
7-11	39.6	5.3	49.4	5.7
12-27	40.6	2.9	50.9	5.5
All ages	39.1	3.1	52.5	5.2

Supplementary Table S.2. Sensitivity of population growth rates to variation in mortality and natality rates across stages of the annual cycle and of the life cycle of satellite/GPS-tagged Black kites.

Ageclass (years)	Sensitivity values				
	Mortality				Breeding success
	Breeding grounds	Southward migration	Wintering stage	Northward migration	
1	0.068	0.074	0.211	0.063	0.008
2-6	0.300	0.297	0.296	0.304	0.028
7-11	0.214	0.216	0.184	0.184	0.021
12-27	0.360	0.333	0.331	0.358	0.032
